# Intermittent fasting suppressed splenic CD205+ G‐MDSC accumulation in a murine breast cancer model by attenuating cell trafficking and inducing apoptosis

**DOI:** 10.1002/fsn3.2510

**Published:** 2021-08-05

**Authors:** Chenghao Fu, Yao Lu, Yiwei Zhang, Mingxi Yu, Shiliang Ma, Shuxia Lyu

**Affiliations:** ^1^ College of Food Science Shenyang Agricultural University Shenyang China; ^2^ College of Bioscience and Biotechnology Shenyang Agricultural University Shenyang China; ^3^ College of Animal Science and Veterinary Medicine Shenyang Agricultural University Shenyang China

**Keywords:** antitumor immunity, apoptosis, CD205+ G‐MDSC, CXCR4, intermittent fasting

## Abstract

Immune‐based interventions are the most promising approach for new cancer treatments to achieve long‐term cancer‐free survival. However, the expansion of myeloid‐derived suppression cells (MDSCs) attenuates the therapeutic potential of immunotherapy. We recently showed that CD205+ granulocytic MDSCs (G‐MDSCs), but not T cells, are sensitive to glucose deficiency. Intermittent fasting (IF) may inhibit the growth of malignant cells by reducing serum glucose levels, but little is known regarding the influence of IF on MDSC expansion. Herein, we observed that IF selectively inhibited splenic accumulation of CD205+ G‐MDSCs in a 4T1 and 4T07 transplant murine breast cancer model. The efficiency of IF in suppressing tumor growth was comparable to that of docetaxel. Further examination revealed that CXCR4 expression was concentrated in CD205+ subsets of tumor‐induced G‐MDSCs. Downregulation of CXCR4 correlated with a reduction in CD205+ G‐MDSC trafficking from bone marrow to the spleen under IF treatment. In addition, ex vivo culture assays showed that glucose deficiency and 2‐deoxy‐D‐glucose (2DG) treatment selectively induced massive death of splenic CD205+ G‐MDSCs. Interestingly, 2DG emulated the phenomena of IF selectively suppressing the accumulation of CD205+ G‐MDSCs in the spleen, upregulating cleaved caspase 3 in the tumor, downregulating Ki67 in the lung, and retarding the growth of transplanted 4T1 and 4T07 murine breast tumors. These findings suggest that IF inhibited cell trafficking through the downregulation of CXCR4 and induced apoptosis by altering glucose metabolism; this, suppressed the accumulation of tumor‐induced splenic CD205+ G‐MDSCs and in turn enhanced antitumor immunity.

## Graphical abstract

We observed that intermittent fasting not only limits the growth of breast tumors but also inhibits the accumulation of immunosuppressive cells, suggesting that intermittent fasting has the potential to enhance the immune system of cancer hosts and is an adjuvant treatment option to improve the effectiveness of cancer immunotherapy.

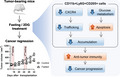

## 1 INTRODUCTION

Myeloid‐derived suppressor cells (MDSCs) are a heterogeneous population of immature cells of myeloid origin (Gabrilovich & Nagaraj, 2009). MDSCs considerably expand during pathological situations, such as cancer, inflammation, and infection (Lv et al., 2019). Two subsets of tumor‐induced MDSCs have been identified based on morphology and cell surface expression of specific molecules. The granulocytic (G‐MDSC) [or polymorphonuclear (PMN‐MDSC)] subset has a CD11b+Ly6G+Ly6Clow phenotype, and the monocytic (M‐MDSC) subset has a CD11b+Ly6G‐Ly6Chigh phenotype (Bronte et al., 2016). MDSCs mediate immunosuppression by perturbing lymphocyte homing, inducing T‐cell anergy, and controlling regulatory T‐cell differentiation. MDCSs have also been implicated in nonimmunological functions, such as promoting angiogenetic tumor cell invasion and metastasis (Priceman et al., 2010; Yang et al., 2004, 2008). Accordingly, MDSCs are broadly viewed as a crucial factor attenuating the efficacy of some cancer immunotherapies, and thus, any interventive strategy that minimizes the negative influence of MDSCs may significantly improve outcomes for patients treated with immunotherapies (Ding et al., 2014; Garritson et al., 2020; Highfill et al., 2014).

The concept of dietary modulation to reduce cytotoxic effects and improve the response to cancer therapy is extremely attractive to many patients suffering intolerable side effects (de Groot et al., 2019; Tajan & Vousden, 2020). Compared with numerous chronic dietary interventions (Brandhorst & Longo, 2019), intermittent fasting (IF) (de Cabo & Mattson, 2019) induces a wide range of changes associated with potent antitumor effects that would be difficult to achieve with even a cocktail of anticancer drugs (de Groot et al., 2019; Lee & Longo, 2011; Nencioni et al., 2018). Hallmarks of a systemic response to fasting in mammals include low levels of glucose, insulin, insulin‐like growth factor 1 (IGF‐1), and leptin and high levels of glucagon, ketone bodies, and adiponectin (Brandhorst et al., 2015; Nencioni et al., 2018). At the molecular level, fasting reduces intracellular signaling cascades (including IGF1R–AKT–mTOR–S6K and cAMP–PKA signaling), increases autophagy, helps normal cells withstand stress, and promotes anticancer immunity (Nencioni et al., 2018; Zhao et al., 2021). There is strong evidence that among the changes associated with fasting, the ones mediating the beneficial anticancer effects are the reduction in IGF‐1 and glucose levels (Lee & Longo, 2011; Lee et al., 2012).

Cancer cells are vulnerable to nutrient deprivation and dependent on specific metabolites (Hanahan & Weinberg, 2011). In most cell types, including malignant cells, IGF‐1 plays a crucial role in glucose uptake and metabolism (Aguirre et al., 2016). Therefore, interventions targeting glucose metabolism are particularly promising (Turbitt et al., 2019). However, immune cells also rely on glycolysis to sustain their clonal expansion and function (Kedia‐Mehta & Finlay, 2019; Leone & Powell, 2020). As a result, it remains unclear whether glucose‐modulating therapies would support or hinder antitumor immunity. We recently showed that CD205+ G‐MDSCs, but not T cells, are sensitive to glucose deficiency (Fu et al., 2020). Therefore, we believe that fasting could potentially enhance the efficacy of cancer immunotherapy if it suppressed the development of MDSCs. In the current study, we evaluated the development of G‐MDSC subpopulations under conditions of fasting and docetaxel treatment using a murine 4T1 and 4T07 transplant breast cancer model. We found that fasting decreased the accumulation of CD205+ G‐MDSCs in the spleens of the mice and did so by inhibiting cell trafficking and glucose metabolism.

## 2 MATERIALS AND METHODS

### 2.1 Animals

Female BALB/c mice (SPF grade, weigh 18 ± 2 g, aged 8–12 weeks) were obtained from the Changsheng Animal Resources Center (Benxi, Liaoning, China). The mice were maintained on a specific pathogen‐free condition with 12 hr day/light cycles.

### 2.2 Cancer cell lines and tumor cell injection

The cancer cell culture was done as previously described (Fu et al., 2020). Briefly, the 4T1 and 4T07 murine breast cancer cell lines were purchased from the Cell Bank of the Chinese Academy of Sciences (Shanghai, China). The cells were cultured in an RPMI1640‐based medium at 37°C and 5% CO_2_.

To obtain a transplanted tumor model, a total number of 2 × 10^5^ 4T1 or 4T07 breast cancer cells were injected subcutaneously into the mouse fat pad of the fourth mammary gland, respectively. The tumor volume was calculated as follows (Euhus et al., 1986): tumor volume (mm^3^) = (length 3 width 3 height)/2, where the height, length, and width are in millimeters. Besides, all tissues were collected after euthanization by pentobarbital injection.

### 2.3 In vivo fasting, 2DG, and docetaxel treatment

The 4T1 and 4T07 tumor‐bearing mice underwent complete food deprivation (fasting) with free access to water for a total of 48 hr. To minimize animal pain, 20% of the bodyweight loss is allowed at most in fasted mice. During fasting treatment, mice were individually housed in a clean new cage to reduce cannibalism, coprophagy, and residual chow. 2‐Deoxy‐D‐glucose (2DG) and docetaxel were obtained from SIGMA (Merck Millipore, Darmstadt, Germany). 15 μg/20 g 2DG and 15 mg/kg docetaxel were injected intraperitoneally into mice at indicated time points.

### 2.4 Ex vivo splenic cells culturation

The ex vivo splenic cell culture was done as previously described (Fu et al., 2020). Briefly, the spleen cells were harvested in a sterile environment. After suspending in glucose‐free DMEM‐based medium (DMEM; Gibco, Grand Island, NY, USA) and RPMI 1,640 medium, the splenic‐single‐cells were plated at a density of 1 × 10^6^ well^−1^ in 24‐well plates. After incubation at 37°C under 5% CO_2_ for 12 hr, cells were washed twice with PBS and detected by flow cytometry (FCM).

### 2.5 Antibodies and flow cytometry

The preparation of single‐cell suspensions was described previously (Fu et al., 2020). Antibodies specific for the following surface markers were used: Ly6G (1A8‐Ly6g), CD11b (M1/70), CD45 (30‐F11), CD205 (205yekta), and TLR2 (CB225) were purchased from eBioscience (San Diego, CA, USA); CCR3 (J073E5), CCR4 (2G12), CCR5 (HM‐CCR5), CCR6 (29‐2L17), CCR7 (4B12), CCR9 (CW‐1.2), CXCR2 (SA203G11), CXCR3 (CXCR3‐173), CXCR4 (L276F12), CXCR5 (L138D7), CXCR7 (8F11‐M16), and CX3CR1(SA011F11) were purchased from Biolegend (San Diego, CA, USA); CCR1 (R&D), CCR2 (R&D), CCR8 (1055C, R&D), CCR10 (R&D), CXCR1 (R&D), and CXCR6 (R&D) were purchased from R&D Systems (Minneapolis, MN, USA). All antibodies were used at the fold dilution described on the manufacturer's recommendation. Unstained cells, single stain, fluorescence minus one control, and isotype controls were used for setting laser voltages and for compensation. FCM data were obtained by FACSAria III (BD Biosciences, San Jose, CA, USA) and analyzed with FlowJo v. 10 (BD Biosciences, San Jose, CA, USA).

### 2.6 Apoptosis assay

An Annexin V/7‐AAD apoptosis detection kit was used to detect the apoptotic cells. Briefly, after cell surface marker staining, cells were sequentially stained by Pacific Blue Annexin V and 7‐AAD. FCM analysis should be done in 15 min after 7‐AAD staining. In addition, above Annexin V‐positive gates, the 7‐AAD‐negative and 7‐AAD‐positive cells were defined as early‐apoptotic cells and late‐apoptotic cells, respectively.

### 2.7 Immunohistochemistry (IHC)

The IHC was done as previously described (Zheng et al., 2020). Briefly, tissues obtained from mice were paraffin‐embedded, fixed, and sectioned. The sections were soaked in 100% ethanol for 10 min twice and then hydrated sequentially through 95% ethanol, 80% ethanol, and distilled water. Before blocking the non‐specific binding via 5% bovine serum albumin, antigen retrieval was performed by microwaving in sodium citrate (pH 6.0). After that, primary antibodies were incubated overnight at 4°C. The secondary antibodies conjugated with HRP were then incubated for 1 hr and rinsed in PBS three times. At last, the sections were cover‐slipped and then sealed before imaging under a microscope (Nikon, Japan).

### 2.8 Statistical analysis

All experiments were repeated at least thrice. During data collection and analysis, the investigator was blinded to the experimental group. Only representative data are exhibited. GraphPad Prism v. 6 (GraphPad Software, Inc., La Jolla, CA, USA) and Microsoft Excel 2016 were used to perform statistical analysis.

Differences between groups were examined using one‐ or two‐way ANOVA or Student's *t* tests. Graphed data represent ≥3 independent experiments and are shown as means ± standard deviation. Unless otherwise indicated, significance was as follows: ***p < .001, **p < .01, and *p < .05.

## 3 RESULTS

### 3.1 Fasting restricted the growth of 4T1 and 4T07 tumors in vivo

**FIGURE 1:**
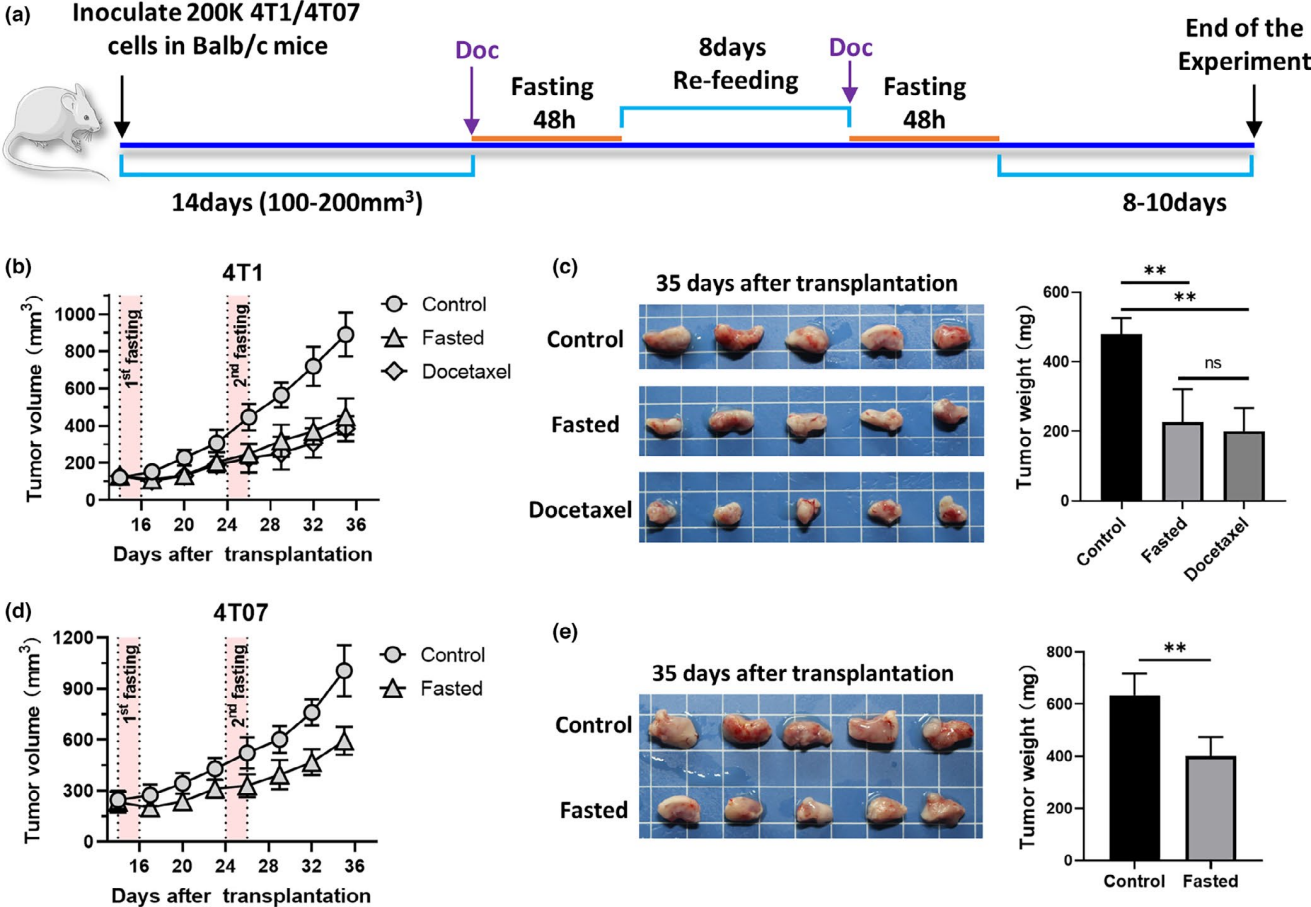
Fasting retarded the growth of 4T1 and 4T07 tumor in vivo. Tumor‐bearing mice were euthanized after fasting or docetaxel treatment. (a) Schematic diagram of the study design. (b and d) Tumor growth curve after fasting or docetaxel treatment in 4T1 and 4T07 BALB/c tumor model. (c and e) Tumor weight of 4T1 and 4T07 tumor after fasting or docetaxel treatment. **p < .01, *p < .05, ns: no statistical significance, and Doc: means docetaxel treatment

In this study, BALB/c female mice were subcutaneously inoculated with an equal number of either 4T1 or 4T07 cells, or without cells. To maintain initial tumor‐size homogeneity, only mice with similar tumor volumes were used. As two cycles of fasting treatment (48‐hr fasting followed by a 8‐day refeeding) (Di Biase et al., 2016; Lee et al., 2012) have been shown to be effective in retarding the 4T1 breast tumor growth, the current experiments were also performed using the two cycles of fasting (Figure 1a). The 4T1 and 4T07 transplanted tumor volumes of fasted groups at the end of the first fasting cycles were significantly smaller than those of the control groups (Figure 1b). After the second fasting cycle, the volume of the 4T1 tumor was less than half of that in the control group (Figure 1c). Similar suppressive effects on tumor growth were observed in the 4T07 breast tumor model (Figure 1d and 1e). The results demonstrate that intermittent fasting treatment has potent suppressive activity on 4T1 and 4T07 breast cancer.

### 3.2 Fasting reduced splenic CD11b+Ly6G+CD205+ cell accumulation in the breast cancer model

**FIGURE 2:**
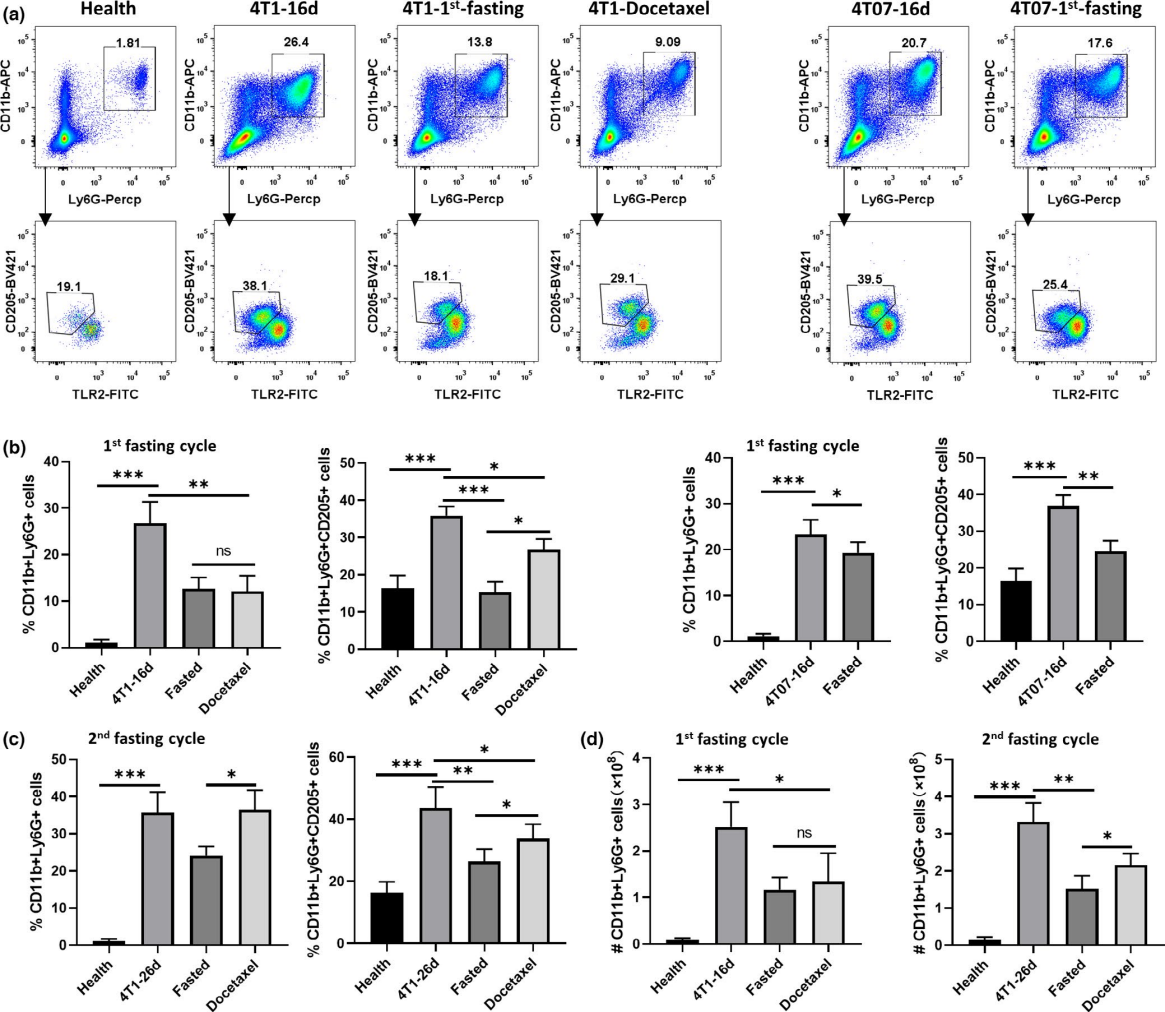
Fasting reduced CD11b+Ly6G+CD205+ cells accumulation in the spleen of breast cancer model. Tumor‐bearing mice were euthanized after fasting or docetaxel treatment, and splenic cells were collected for FCM analysis. (a) FCM plots are shown for representative CD11b+Ly6G+ and CD11b+ Ly6G+ CD205+ cells variation. (b, c) The ratio of CD11b+Ly6G+ and CD11b+Ly6G+CD205+ cells after 1st and 2nd fasting cycle representatively. (d) The total cells number of splenic CD11b+ Ly6G+ and CD11b+ Ly6G+ CD205+ cells. Note: CD11b+Ly6G+ cell was pre‐gated on 7‐AAD‐CD45+ gates with Flowjo software. ***p < .001, **p < .01, and *p < .05. 1^st^‐ or 2^nd^‐ fasting means tumor‐bearing mice suffering the first or second fasting treatment

We next examined the accumulation of CD11b+Ly6G+CD205+ cells in vivo in fasting treated tumor‐bearing mice. The mice were euthanized, and their spleens were collected for FCM detection after a 48‐hr fasting period. Our results showed that, in healthy mouse spleens, <2% of all splenic CD45+ cells were CD11b+Ly6G+ cells, and approximately 20% of CD11b+Ly6G+ cells were CD205+ (Figure 2a and b). The transplanted 4T1 tumors and 4T07 tumors induced a 10‐fold increase in the accumulation of CD11b+Ly6G+ cells (Figure 2a and d). In addition, in 4T1 and 4T07 tumor‐bearing mice, the proportion of CD11b+Ly6G+CD205+ cells doubled compared with that in the healthy mice (Figure 2a). Furthermore, in 4T1 tumor‐bearing mice, fasting decreased the proportion of CD11b+Ly6G+CD205+ cells by at least half. Similar results were observed in the 4T07 tumor‐bearing mice (Figure 2a and d). In the 4T1 tumor‐bearing mice, docetaxel also suppressed the accumulation of CD11b+Ly6G+ cells; however, the difference is that fasting selectively suppressed the CD205+ subsets of CD11b+Ly6G+ cells. Moreover, the suppression effects on CD11b+Ly6G+CD205+ cells remained after the second fasting cycle (Figure 2c).

### 3.3 Fasting attenuated splenic CD11b+ Ly6G+ CD205+ cell accumulation and CXCR4 expression

**FIGURE 3:**
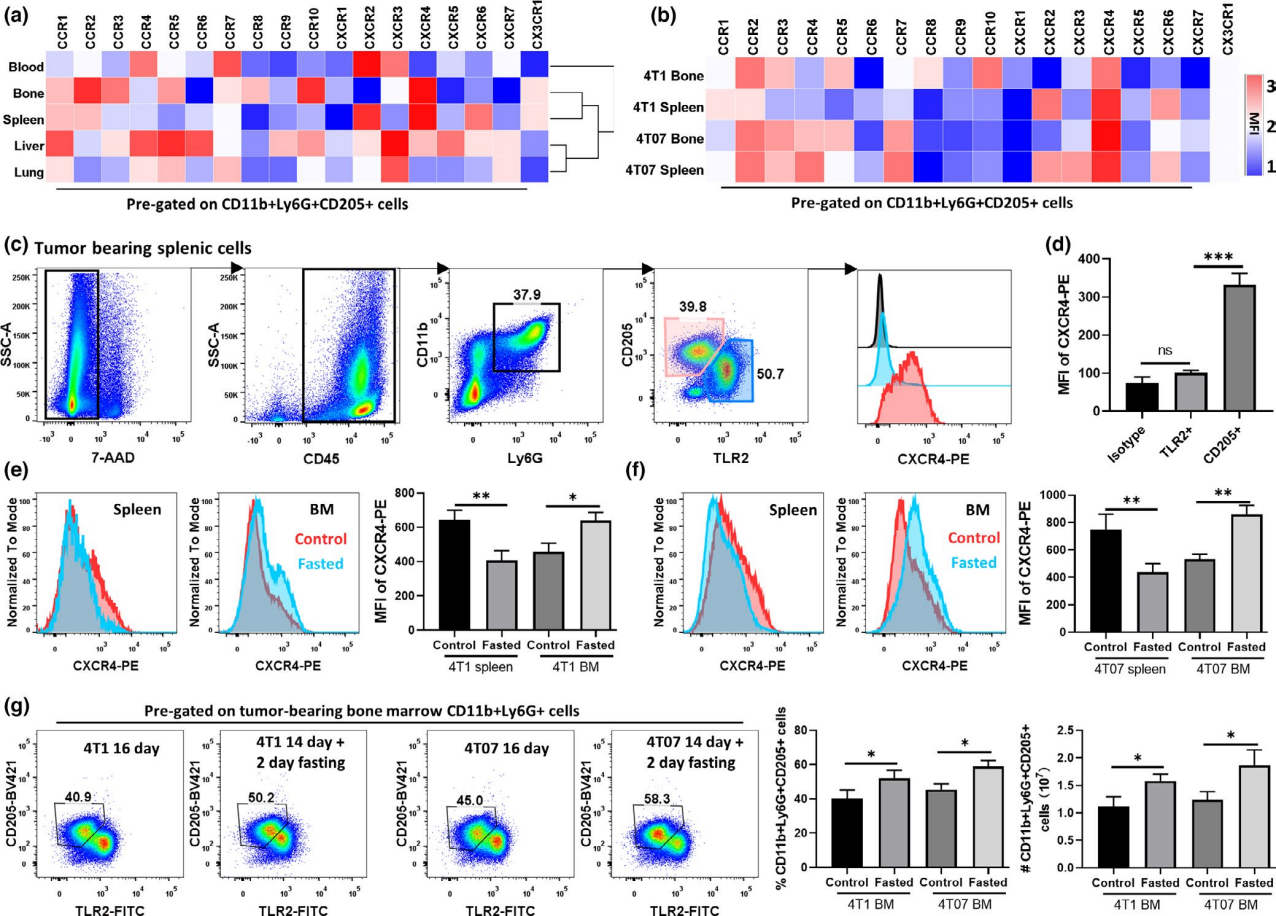
Fasting attenuated the accumulation and CXCR4 expression of splenic CD11b+ Ly6G+ CD205+ cells. Tumor‐bearing mice were euthanized, and spleen and bone marrow (BM) cells were collected for FCM analysis. (a, b) Heatmap shows the mean fluorescence intensity (MFI) of chemokine receptors on CD11b+Ly6G+CD205+ cells detected by FCM. (c) Gating strategy of CXCR4 fluorescence intensity in CD205+ (CD11b+Ly6G+ cells, red) and TLR2+ (CD11b+Ly6G+ cells, blue) subpopulation in the spleen. The isotype of CXCR4 is rat IgG 2b, Kapa (black). (d) MFI of CD205+ and TLR2+ G‐MDSC subpopulation in the spleen. (e–g) Spleen and BM cells were collected from tumor‐bearing mice after fasting treatment. (e, f) MFI of CXCR4 in spleen and BM CD205+G‐MDSC in 4T1‐(e) and 4T07‐(f) tumor model. (g) Proportion and number of BM‐CD205+G‐MDSC in 4T1 and 4T07 tumor model. ***p < .001, **p < .01, and *p < .05

To determine whether chemokine receptors are involved in fasting‐induced MDSC reduction in the spleen, we employed FCM to evaluate the expression of 18 chemokine receptors on CD11b+ Ly6G+ CD205+ cells (Figure 3a and b). We observed that CD11b+Ly6G+CD205+ cells in the bone marrow and spleen showed similar chemokine receptor expression profiles. CD11b+Ly6G+CD205+ cells from the liver, spleen, and bone marrow specifically expressed CXCR4 (Figure 3a). In the bone marrow and spleen of 4T07 tumor‐bearing mice, CD11b+ Ly6G+ CD205+ cells also expressed high levels of CXCR4 (Figure 3b). In addition, the mean fluorescence intensity (MFI) of CXCR4 in TLR2+ G‐MDSCs was comparable to that of the isotype control (Figure 3c). In contrast, the MFI of CXCR4 in CD205+ G‐MDSCs was threefold higher than that of TLR2+ G‐MDSCs (Figure 3d).

The variation of CXCR4 expression levels was then assessed after fasting treatment. Interestingly, in the splenic CD205+G‐MDSC from 4T1 and 4T07 tumor‐bearing mice, the MFI of CXCR4 significantly decreased after 48‐hr fasting; In contrast, CD205+ G‐MDSCs from bone marrow expressed increased levels of CXCR4 (Figure 3e and f). Furthermore, in 4T1 and 4T07 tumor‐bearing mice treated with 48‐hr fasting, the quantity and proportion of CD205+G‐MDSCs increased markedly compared with that of control groups (Figure 3g).

### 3.4 2DG and glucose deficiency specifically induced CD11b+Ly6G+CD205+ cell apoptosis

**FIGURE 4:**
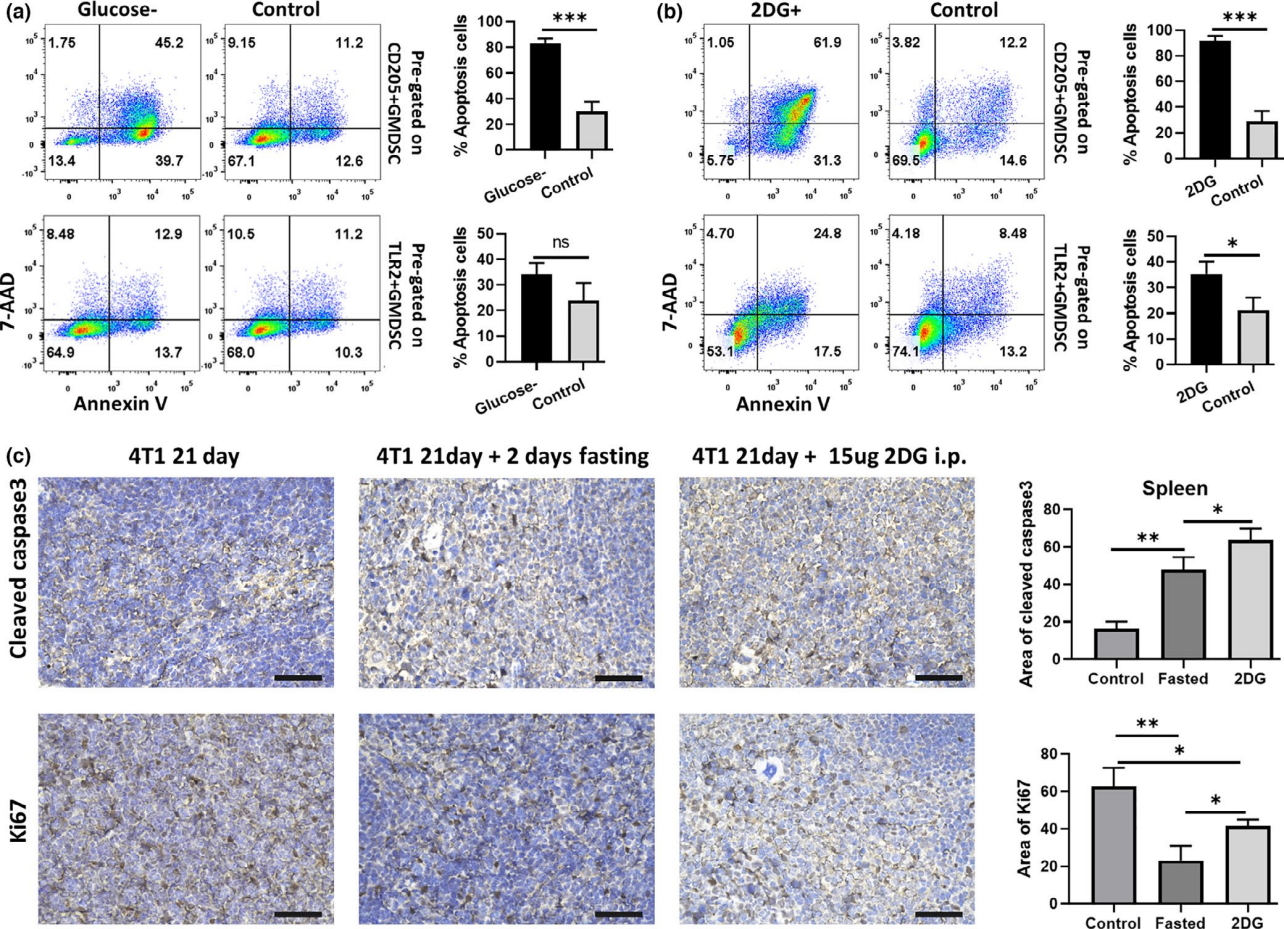
2dG and glucose deficiency specifically induced CD11b+Ly6G+CD205+ cells apoptosis. (a, b) The spleen cells were harvested from tumor‐bearing mice and then detected by Annexin V and 7‐AAD assay after ex vivo culture. (a) Cell apoptosis rate after glucose deficiency culture. 'glucose‐' or '2DG+': 'no glucose' or 'added 2DG' in the medium. (b) Cell apoptosis rate after cultured in RPMI 1,640 medium and added 2DG (2DG+) or not (Control), FCM detection was performed 12 hr postculture. (c) Tumor‐bearing mice were euthanized after fasting or 2DG treatment, and the spleen was collected for IHC detection. 'i.p.': intraperitoneal injection. The representative cleaved caspase 3 and Ki67 IHC staining in the spleen are shown. Scale = 100 μm. ***p < .001, **p < .01, and *p < .05

We next examined the apoptosis rate of CD205+G‐MDSC in glucose restriction environment which mimicking to that of fasted mice. The results show that more than 65% of CD205+ G‐MDSCs were annexin V and 7AAD negative after 12 hr of ex vivo culture, whereas that proportion decreased to 13% when there was no glucose in the medium. Similar changes were not observed in the TLR2+G‐MDSC ex vivo culture assay (Figure 4a). Interestingly, when treated with 2DG, we observed that less than 6% of ex vivo cultured CD205+ G‐MDSCs survived. In contrast, 2DG treatment induced apoptosis in only a small proportion of TLR2+ G‐MDSCs in ex vivo culture (Figure 4b). These results suggest that glucose deficiency greatly increased apoptosis of the CD205+ G‐MDSCs.

Furthermore, we checked the cell apoptosis and proliferation markers via immunohistochemistry (IHC) in fasting and 2DG‐treated 4T1 tumor‐bearing mice. The results showed significantly higher levels of cleaved caspase 3 and lower levels of Ki67 staining in the spleens of fasting and 2DG‐treated tumor‐bearing mice compared with those of the control groups (Figure 4c). Moreover, 2DG treatment induced higher in vivo levels of apoptotic features than those in the control groups, which different from results of the MDSC ex vivo culture assays.

### 3.5 2DG prevented CD205+ G‐MDSC accumulation and retarded tumor progression

**FIGURE 5:**
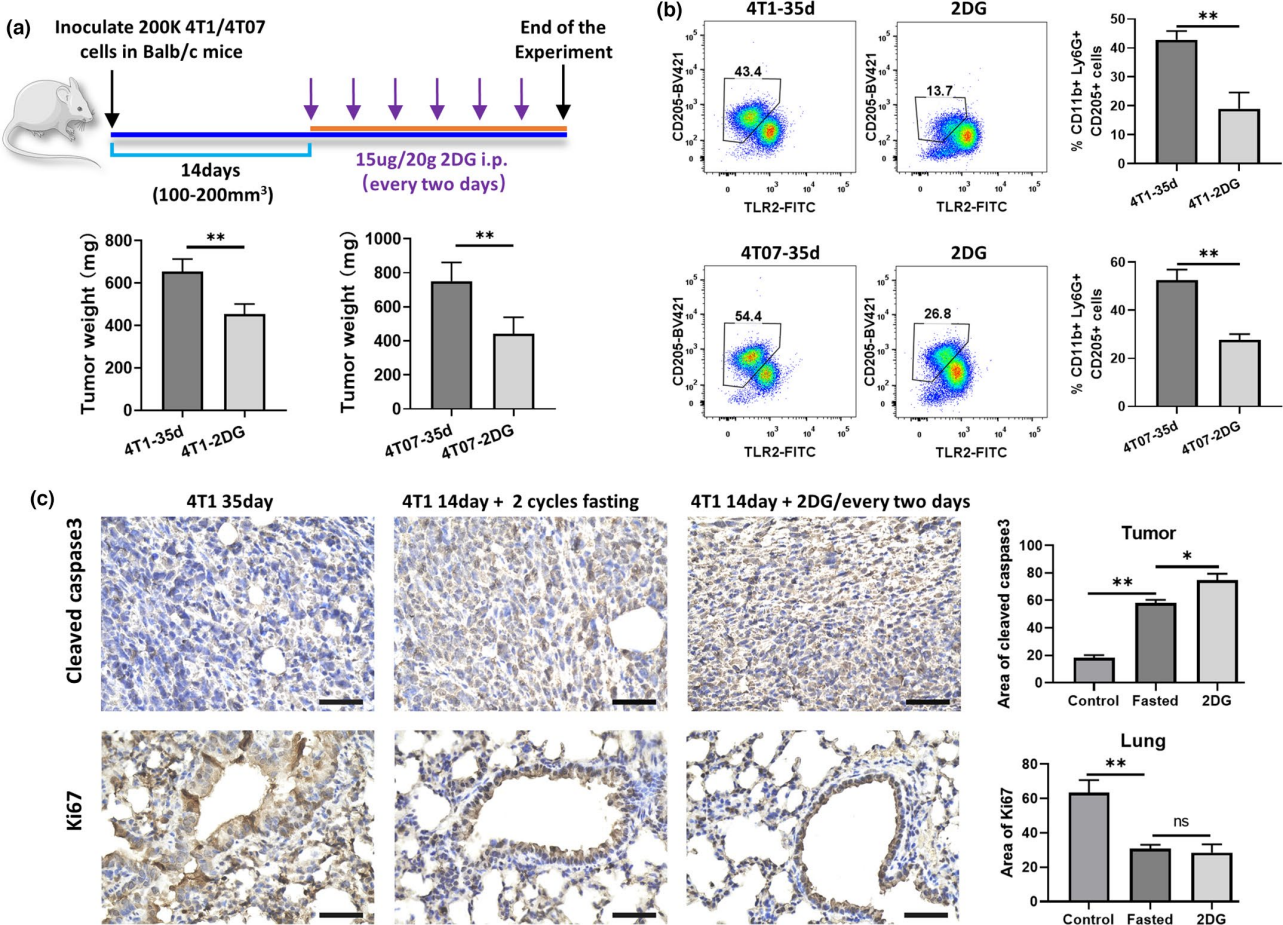
2dG prevented the accumulation of CD205+G‐MDSC and retarded the tumor progression. Tumor‐bearing mice were euthanized after fasting or 2DG treatment, and the spleen was collected for FCM and IHC detection. (a) Tumor weight of 4T1 or 4T07 tumor model with or without 2DG treatment. (b) Percentage of splenic CD11b+ Ly6G+ CD205+ cell in 4T1 or 4T07 tumor model with or without 2DG treatment. (c) The representative cleaved caspase 3 and Ki67 IHC staining in the tumor and lung are shown. Scale = 100 μm. ***p < .001, **p < .01, and *p < .05

We next assessed the correlation between CD205+ G‐MDSC reduction and tumor growth rate of 2DG treatment in vivo. Fourteen days after tumor cell transplantation, 2DG was injected intraperitoneally every other day, and six times injection in total (Figure 5a). The results showed that the tumor weight of 2DG‐treated groups was about two‐thirds that of the control groups in both the 4T1 and 4T07 tumor‐bearing mice. Consistently, 60% and 50% decrease in the proportion of splenic CD205+ G‐MDSCs was observed in 2DG‐treated 4T1 and 4T07 tumor‐bearing mice, respectively. These results suggested that the CD205+G‐MDSC reduction can induced by 2DG treatment.

In addition, IHC results showed that the area of cleaved caspase 3 staining was approximately 75% of the tumor mass in the 2DG‐treated 4T1 tumor model, which was more than three times that in the control groups (Figure 5c). Furthermore, in the lung control groups, Ki67 staining was approximately 60%, which was more than two times that of the fasting and 2DG treatment groups (Figure 5c). These results indicated that fasting induced a reduction in CD205+ G‐MDSC accumulation, at least in part, through the regulation of glucose restriction.

## 4 DISCUSSION

New interventions that can delay tumor progression or elevate antitumor effects of the immune system have drawn our attention in recent years. On the one hand, active ingredients extracted from natural foods might have potent antitumor efficacy and presented a rich source of structural information about biomolecules and their complexes (Martorell et al., 2017; Zheng et al., 2019). On the other hand, the concept of dietary modulation to reduce cytotoxic effects and improve the response to cancer therapy represented by IF is extremely appealing to many patients (de Groot et al., 2019; Tajan & Vousden, 2020). A series of research reviewed by Lee and colleagues showed that the protective effect of fasting in mammals is mediated, in part, by a greater than 50% reduction in glucose and IGF‐1 levels (Lee & Longo, 2011). Furthermore, multiple cycles of fasting were as effective as chemotherapeutic agents in delaying the progression of different tumors (Lee et al., 2012). This is consistent with our IF treatment results, yielding approximately a 50% reduction in 4T1 tumor weight, higher than that in 4T07 tumors (with approximately a 30% reduction, Figure 1b and c).

The expansion of G‐MDSCs is one of the main obstacles to improved antitumor immunity mediated by immune‐based interventions (Zhou et al., 2018). In the current study, we showed that IF treatment inhibited both 4T1 and 4T07 tumor progression and, at the same time, reduced total G‐MDSC accumulation (Figure 2 and Figure 3). Given that G‐MDSC can mediate immune suppression through both antigen‐specific T‐cell suppression, such as ROS production, and non‐specific mechanisms, such as the production of arginase 1, interleukin‐10, and cyclooxygenase‐2 (Bronte et al., 2016; Zhou et al., 2018), we believe that IF can also increase the antitumor effects of T cells in the cancer host. In fact, evidence shows that fasting‐mimicking diets can downregulate heme oxygenase 1 (HO1) expression in breast cancer cells, making them more susceptible to CD8+ cytotoxic T cells, possibly by countering the immunosuppressive effect of regulatory T (Treg) cells (Di Biase et al., 2016; de Groot et al., 2019). In summary, those observations indicate that IF improved the tumor‐induced imbalance of antitumor immunity.

Furthermore, we revealed that fasting selectively suppressed the accumulation of CD205+ G‐MDSCs, which was different from the docetaxel treatment (Figure 2a). Evidence shows that docetaxel is not only a cytotoxic chemotherapy drug, but also has a chemo‐immunomodulating property as a therapeutic intervention for cancer (Fleming et al., 2018; Kodumudi et al., 2010). Docetaxel can selectively eliminate MDSCs and enhance antitumor immunity responses (Alizadeh et al., 2014; Millrud et al., 2018). Those observations suggest that fasting modulated the tumor‐induced imbalance of antitumor immunity via a mechanism different from that of docetaxel. Moreover, chemokine receptors act as responders to chemoattractants to guide the migration of cells. Evidence shows that CXCR2 mediates G‐MDSC tumor trafficking in murine rhabdomyosarcoma models. CXCR2 deficiency prevents MDSC trafficking and enhances anti‐PD1 efficacy (Highfill et al., 2014). Conversely, CXCR2 and CXCR4 antagonistically regulate Gr‐1+SSC‐high neutrophil trafficking from murine bone marrow (Eash et al., 2010). Herein, we show that CXCR4 expression was concentrated in CD205+ G‐MDSCs, which are also characterized as Gr‐1+ SSC‐high (Fu et al., 2020). Downregulation of CXCR4 correlated with a reduction in CD205+ G‐MDSC trafficking from bone marrow to the spleen under IF treatment. Therefore, we suggest that the CXCR4 axis is a candidate mediator of fasting‐induced CD205+ G‐MDSC reduction in breast cancer hosts.

Recently, we showed that transplanted 4T1 and 4T07 tumors induce CD205+ G‐MDSC expansion in the spleen and liver, but not EMT6 tumors (Fu et al., 2020). Interestingly, in the current study we observed that both 2DG and fasting retarded tumor growth of transplanted 4T1 and 4T07 cells (Figure 5a). However, 2DG combined with fasting does not suppress transplanted EMT6 tumor growth, despite 2DG inhibiting the viability of EMT6 cells in vitro (Aft et al., 2003; Rockwell & Schulz, 1984). Herein, we also revealed that 2DG treatment selectively induced massive death of splenic CD205+ G‐MDSCs (Figure 4b), and it could also emulate the phenomena of IF selectively suppressing the accumulation of CD205+ G‐MDSCs in the spleen (Figure 5b). We know that 4T1 tumor cells develop overt metastasis in vivo, while 4T07 and EMT6 tumor‐bearing mice fail to generate spontaneous metastatic tumors, despite their capacity to disseminate into secondary organs (Aslakson & Miller, 1992; Piranlioglu et al., 2019). These observations suggest that CD205+ G‐MDSC might play an important role in cancer metastasis. Moreover, 2DG is a glucose molecule with the 2‐hydroxyl group replaced by hydrogen; thus, it cannot undergo further glycolysis. Therefore, 2DG is usually used as a glucose metabolism inhibitor in cancer research (Zhang et al., 2014). These results indicated that CD205+ G‐MDSC apoptosis was induced by IF, at least in part, through the regulation of glucose metabolism.

## 5 CONCLUSION

In conclusion, this study revealed that IF has suppressive effects on a certain subpopulation of MDSC (CD205+ G‐MDSC) in 4T1 and 4T07 murine tumor models. We further demonstrated that IF suppressed splenic CD205+ G‐MDSC accumulation in a murine breast cancer model by attenuating cell trafficking and inducing apoptosis. Furthermore, the results showed that IF inhibited cell trafficking through the downregulation of CXCR4 and induced apoptosis by altering glucose metabolism and in turn enhanced antitumor immunity. However, further studies to examine the antitumor activity of T cells are needed to assess the overall antitumor efficacy of IF.

## CONFLICT OF INTEREST

The authors have no conflict of interest to declare.

## AUTHOR CONTRIBUTIONS

**Chenghao Fu:** Conceptualization (equal); Data curation (equal); Investigation (equal); Methodology (equal); Resources (equal); Visualization (equal); Writing‐original draft (equal); Writing‐review & editing (equal). **Yao Lu:** Data curation (equal); Formal analysis (equal); Investigation (equal); Methodology (equal); Software (equal). **Yiwei Zhang:** Data curation (equal); Formal analysis (equal); Methodology (equal); Validation (equal). **Mingxi Yu:** Conceptualization (equal); Formal analysis (equal); Methodology (equal). **Shiliang Ma:** Conceptualization (equal); Funding acquisition (equal); Methodology (equal); Supervision (equal); Validation (equal); Writing‐review & editing (equal). **Shuxia Lyu:** Conceptualization (equal); Project administration (equal); Supervision (equal); Validation (equal); Visualization (equal); Writing‐review & editing (equal).

## ETHICAL APPROVAL

All mice used in this study were cared in accordance with the Animal Husbandry Guidelines of Shenyang Agricultural University (SYAU). The laboratory animal experiments were approved by the Ethical Committee of SYAU (Permit No. SYXK <Liao > 2011‐0001).

## Data Availability

All data generated or analyzed during this study are included in this published article.
